# A matched comparison of revision rates of cemented Oxford Unicompartmental Knee Replacements with Single and Twin Peg femoral components, based on data from the National Joint Registry for England, Wales, Northern Ireland and the Isle of Man

**DOI:** 10.1080/17453674.2020.1748288

**Published:** 2020-04-16

**Authors:** Hasan R Mohammad, Gulraj S Matharu, Andrew Judge, David W Murray

**Affiliations:** a Nuffield Department of Orthopaedics, Rheumatology and Musculoskeletal Sciences University of Oxford, Nuffield Orthopaedic Centre, Oxford, UK;;; b Musculoskeletal Research Unit, Bristol Medical School, University of Bristol, Bristol, UK

## Abstract

Background and purpose — Registries report high revision rates after unicompartmental knee replacement (UKR) due, in part, to aseptic loosing. In an attempt to improve Oxford UKR femoral component fixation a new design was introduced with a Twin rather than a Single peg. We used the National Joint Registry (NJR) to compare the 5-year outcomes of the Single and Twin Peg cemented Oxford UKRs.

Patients and methods — We performed a retrospective observational study using NJR data on propensity score matched Single and Twin Peg UKRs (matched for patient, implant and surgical factors). Data on 2,834 Single Peg and 2,834 Twin Peg were analyzed. Cumulative implant survival was calculated using the Kaplan–Meier method and comparisons between groups performed using Cox regression models.

Results — In the matched cohort, the mean follow up for both Single and Twin Peg UKRs was 3.3 (SD 2) and 3.4 years (SD 2) respectively. The 5-year cumulative implant survival rates for Single Peg and Twin Peg were 94.8% (95% CI 93.6–95.8) and 96.2% (CI 95.1–97.1) respectively. Implant revision rates were statistically significantly lower in the Twin Peg (hazard ratio [HR)] = 0.74; p = 0.04). The revision rate for femoral component aseptic loosening decreased significantly (p = 0.03) from 0.4% (n = 11) with the Single Peg to 0.1% (n = 3) with the Twin Peg. The revision rate for pain decreased significantly (p = 0.01) from 0.8% (n = 23) with the Single Peg to 0.3% (n = 9) with the Twin Peg. No other reasons for revision had significant differences in revision rates.

Interpretation — The revision rate for the cemented Twin Peg Oxford UKR was 26% less than the Single Peg Oxford UKR. This was mainly because the revision rates for femoral loosening and pain more than halved. This suggests that the Twin Peg component should be used in preference to the Single Peg design.

Unicompartmental knee replacement (UKR) for anteromedial knee arthritis has many advantages over total knee replacement (TKR) (Wilson et al. 2019). However, national registries suggest that UKR revision rates are several times higher than TKR, with aseptic loosening a leading cause (New Zealand Joint Registry 2016, National Joint Registry 2018).

The most commonly used UKR is the Phase 3 Oxford UKR. The initial Phase 3 femoral component, like its predecessors, was spherical and cemented. It had a single peg that was thought to be helpful as it allowed the component to seat optimally (Figure 1). However, as 25–50% of aseptic loosening was femoral (Mohammad et al. 2018), it was felt that the introduction of a Twin Peg component that might improve fixation would be advantageous. During surgery a small hole is made in the femur anterior to the main peg hole to stabilize the femoral saw guide. A second peg, which would fit in the small hole, was therefore added, allowing the new component to be used with standard instrumentation. In order to support the peg, the spherical part of the component was extended about 15° further anteriorly. To accommodate this extension more bone is removed anteriorly, which decreases the risk of the bearing impinging This also allows the femoral component to be implanted in increased flexion.

**Figure 1.**
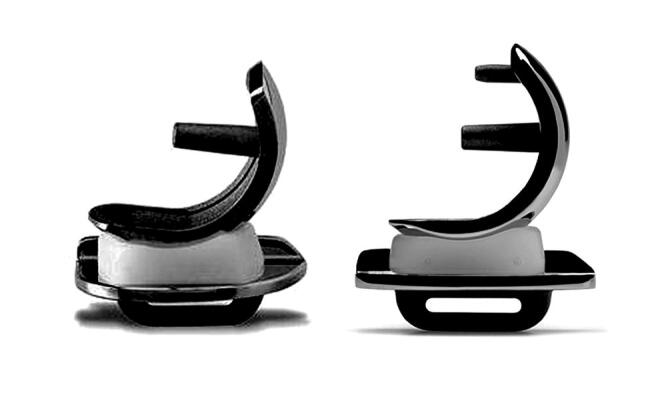
The Oxford UKR with Single and Twin Peg femoral component.

The Twin Peg cemented femoral component was introduced in 2003 but has only been widely used since 2009 (Figure 1) (White et al. 2015). A cementless version of the Twin Peg component was also introduced at a similar time. The Twin Peg cemented component is used with the same cemented tibial component and polyethylene bearing as the Single Peg component. We are not aware of any direct comparative clinical studies of Single Peg and Twin Peg cemented femoral components.

The National Joint Registry for England, Wales, Northern Ireland and Isle of Man (NJR) is the largest replacement registry (National Joint Registry 2018). We used NJR data to compare the revision rate and mechanisms of implant failure, in particular femoral component aseptic loosening, following cemented medial Oxford UKRs using Single and Twin Peg femoral components.

## Patients and methods

We performed a retrospective observational study using the NJR database after NJR Research Sub-Committee approval (National Joint Registry 2018). Data collected by the NJR includes patient, implant, and surgical information. The database has excellent linkability to subsequent revision surgery and is also linked to the Office of National Statistics, which provides mortality data.

**Figure 2.**
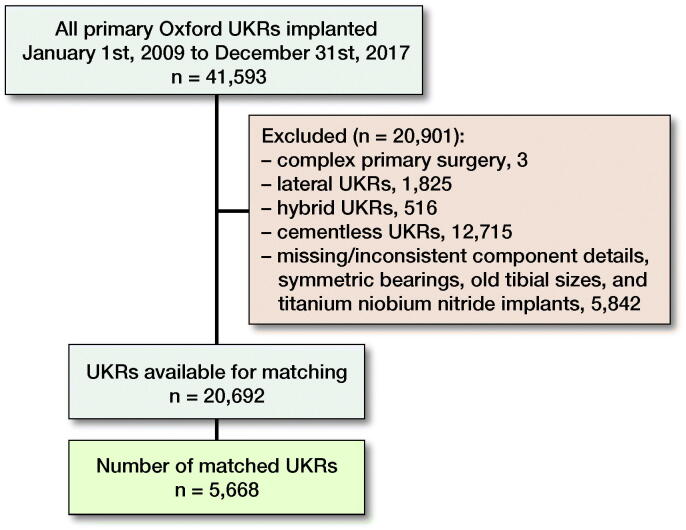
Flowchart of study selection criteria.

Anonymized patient data were extracted from the NJR, which included all primary Oxford UKRs implanted between January 1, 2009 and December 31, 2017 (n = 41,593). After data cleaning there were 20,692 medial cemented Oxford UKRs (17,855 Single Peg and 2,837 Twin Peg) eligible for study inclusion (Figure 2).

The study exposure was the peg design (Single vs. Twin Peg). Given the potential for factors other than peg design to affect the revision rate (Prempeh and Cherry 2008, Selby et al. 2012, Judge et al. 2013, Elmallah et al. 2015, Lim et al. 2015, Bayliss et al. 2017, Hosaka et al. 2017, Picard et al. 2018, Mohammad et al. 2020b) we a priori matched the Single and Twin Peg UKRs for multiple known confounders using propensity scores. Logistic regression was used to generate a propensity score representing the probability that a patient received a Twin Peg UKR. These propensity scores were generated from patient demographics, surgical factors, and implant factors. Specifically, factors used for matching were: age, sex, primary diagnosis, unilateral/bilateral UKRs, ASA grade, chemical thromboprophylaxis, mechanical thromboprophylaxis, year of surgery, operating surgeon grade, surgeon caseload, surgical approach, operating technique and implant component sizes (Table 1). Surgeon caseload was defined as the average number of UKRs done per calendar year by the operating surgeon and stratified into low (< 10 cases/year), medium (10 to < 30 cases/year) and high volume (≥ 30 cases/year) as described previously (Mohammad et al. 2020a). BMI was not used for matching given it had a significant proportion of missing data. However, our data demonstrate that BMI was well balanced between groups and our approach is similar to previous studies using NJR data (Matharu et al. 2017, Mohammad et al. 2020a).

**Table 1.** Patient and surgical factors. Values are number (%) unless otherwise specified

	Unmatched cohort			Matched cohort		
	Single Peg	Twin Peg		Single Peg	Twin Peg	
Covariate	17,855 (86)	2,837 (14)	SMD	2,834 (50)	2,834 (50)	SMD
Sex			0.06			0.009
Female	8,454 (47)	1,431 (50)		1,441 (51)	1,428 (50)	
Male	9,401 (53)	1,406 (50)		1,393 (49)	1,406 (50)	
Age at surgery			0.02			0.01
mean (SD)	65 (10)	65 (10)		65 (10)	65 (10)	0.03
BMI, n	13,159 (	1,868 (	0.04	2,165 (	1,865 (	
mean (SD)	30 (5)	30 (5)		30 (5)	30 (5)	
Primary diagnosis			0.04			0.02
Primary OA	17,676 (99)	2,797 (99)		2,799 (99)	2,794 (99)	
Other	179 (1)	40 (1)		35 (1)	40 (1)	
Bilateral UKRs	469 (3)	44 (2)	0.08	55 (2)	44 (2)	0.03
ASA grade			0.05			0.05
1	3,344 (19)	488 (17)		483 (17)	488 (17)	
2	13,024 (73)	2,082 (73)		2,119 (75)	2,079 (73)	
3 or above	1,487 (8)	267 (10)		232 (8)	267 (10)	
VTEP— chemical			0.4			0.06
LMWH (± other)	10,447 (59)	2,020 (71)		1,972 (70)	2,017 (71)	
Aspirin only	1,426 (8)	208 (7)		240 (8)	208 (7)	
Other	4,731 (26)	590 (21)		593 (21)	590 (21)	
None	1,251 (7)	19 (1)		29 (1)	19 (1)	
VTEP— mechanical			0.08			0.003
Any	17,430 (98)	2,800 (99)		2,798 (99)	2,797 (99)	
None	425 (2)	37 (1)		36 (1)	37 (1)	
Year of surgery			0.7			0.3
2009	2,416 (14)	133 (5)		74 (3)	133 (5)	
2010	2,396 (13)	175 (6)		85 (3)	175 (6)	
2011	2,371 (13)	178 (6)		167 (6)	178 (6)	
2012	2,285 (13)	192 (7)		246 (9)	192 (7)	
2013	2,296 (13)	276 (10)		366 (13)	276 (10)	
2014	2,173 (12)	457 (16)		486 (17)	457 (16)	
2015	1,633 (9)	475 (17)		520 (18)	475 (17)	
2016	1,275 (7)	440 (15)		492 (17)	440 (15)	
2017	1,010 (6)	511 (18)		398 (14)	508 (18)	
Surgeon grade			0.06			0.02
Consultant	16,462 (92)	2,656 (94)		2,668 (94)	2,653 (94)	
Other	1,393 (8)	181 (6)		166 (6)	181 (6)	
Surgeon caseload			0.3			0.04
< 10 cases/year	7,266 (41)	893 (31)		915 (32)	892 (31)	
10–29 cases/year	7,731 (43)	1,104 (39)		1,131 (40)	1,103 (39)	
≥ 30 cases/year	2,858 (16)	840 (30)		788 (28)	839 (30)	
Surgical approach			0.09			0.03
Medial parapatellar	16,283 (91)	2,513 (89)		2,536 (89)	2,511 (89)	
Other	1,572 (9)	324 (11)		298 (11)	323 (11)	
Minimally invasive surgery			0.2			0.01
No	9,171 (51)	1,691 (60)		1,675 (59)	1,689 (60)	
Yes	8,684 (49)	1,146 (40)		1,159 (41)	1,145 (40)	
Femoral component size			0.1			0.02
Extra small	43 (0.2)	6 (0.2)		4 (0.1)	6 (0.2)	
Small	4,076 (23)	796 (28)		788 (28)	793 (28)	
Medium	9,536 (53)	1,451 (51)		1,462 (52)	1,451 (51)	
Large	4,170 (23)	582 (21)		577 (20)	582 (21)	
Extra large	30 (0.2)	2 (0.1)		3 (0.1)	2 (0.1)	
Tibial component size			0.09			0.02
AA	83 (0.5)	11 (0.4)		12 (0.4)	11 (0.4)	
A	2,109 (12)	399 (14)		382 (14)	397 (14)	
B	4,151 (23)	607 (21)		614 (22)	607 (21)	
C	5,071 (28)	798 (28)		809 (29)	798 (28)	
D	4,120 (23)	636 (22)		642 (23)	636 (22)	
E	1,871 (11)	294 (10)		286 (10)	293 (10)	
F	450 (3)	92 (3)		89 (3)	92 (3)	
Bearing size			0.1			0.04
3	3,972 (22)	635 (22)		652 (23)	635 (22)	
4	7,201 (40)	1,163 (41)		1,128 (40)	1,162 (41)	
5	3,786 (21)	623 (22)		617 (22)	622 (22)	
6	1,705 (10)	282 (10)		286 (10)	281 (10)	
7	760 (4)	105 (4)		120 (4)	105 (4)	
8	282 (2)	16 (0.6)		15 (0.5)	16 (0.6)	
9	149 (0.8)	13 (0.5)		16 (0.6)	13 (0.5)	
Bone graft used			0.03			< 0.001
No	17,810 (100)	2,833 (100)		2,830 (100)	2,830 (100)	
Yes	45 (0.3)	4 (0.1)		4 (0.1)	4 (0.1)	

OA = osteoarthritis, SD = standard deviation, SMD = standardized mean difference,

UKR = unicompartmental knee replacement, VTEP = venous thromboembolism prophylaxis.

We matched using a 1:1 ratio on the logit of the propensity score with a 0.02-SD calliper width. We used greedy matching without replacement, which has superior performance for estimating treatment effects (Austin 2009). Standardized mean differences (SMDs) were examined both before and after matching to assess for any covariate imbalance between the different peg design groups, with SMDs of 10% or more considered suggestive of imbalance (Austin 2009). After matching, 5,668 UKRs (2,834 Single Peg and 2,834 Twin Peg UKRs) were included for analysis.

### Statistics

Outcomes of interest were: (1) implant survival and (2) indications for revision surgery, particularly femoral component aseptic loosening. Cumulative survival was determined using the Kaplan–Meier method. The endpoint for implant survival was revision surgery (any addition, removal, or exchange of implant component). Implant survival was compared between the Single and Twin Peg groups, using Cox regression models, with the proportional hazards assumptions assessed and satisfied in all analyses. Additionally, to account for clustering within the matched cohort, a robust variance estimator was used in regression models. Univariable and adjusted models were also assessed. The adjusted models included covariates with residual imbalance after matching (SMD of 10% or more) (Austin 2009). A multi-level frailty model was tested in regression models to adjust for patient clustering within surgeons. The proportional chi-squared test with Yate’s correction or 2-sided Fisher’s exact test was used to compare the indications for revision surgery between groups. The latter was used only when either group had an expected frequency of under 5.

The NJR database allows for revisions for UKRs with any aseptic loosening to be analyzed and also for aseptic loosening by each component involved (e.g., femoral or tibial component). The primary analysis was of revision for aseptic femoral loosening. Aseptic tibial loosening and overall loosening rates were also analyzed as there were some cases of combined tibial and femoral loosening.

All statistical analyses were performed using Stata (Version 15.1; StataCorp, College Station, TX, USA) except propensity score matching, which was performed using R (Version 3.4.0; R Foundation for Statistical Computing, Vienna, Austria). P-values of < 0.05 were considered significant, with 95% confidence intervals (CI) presented.

### Ethics, funding, and potential conflicts of interest

This study was based entirely on existing patient records acquired during routine clinical care and thus did not require ethical approval. This project was fully approved by the NJR Research Sub Committee. Zimmer Biomet provided funding for the research but were not involved in the study.

## Results

The matched cohort included 5,668 Oxford UKRs, with 2,834 Single Peg UKRs and 2,834 Twin Peg UKRs. The mean age at surgery was 65 years (SD 10), with 51% of the cohort being female. The mean BMI was 30 (SD 5) with the primary indication for surgery being osteoarthritis in 99%.

Patient, surgical, and implant characteristics became well balanced between the Single Peg and Twin Peg groups after propensity score matching (Table 1). The only covariate with residual imbalance was year of primary surgery, which when adjusted for in the regression models did not change the findings and is presented below.

**Figure 3.**
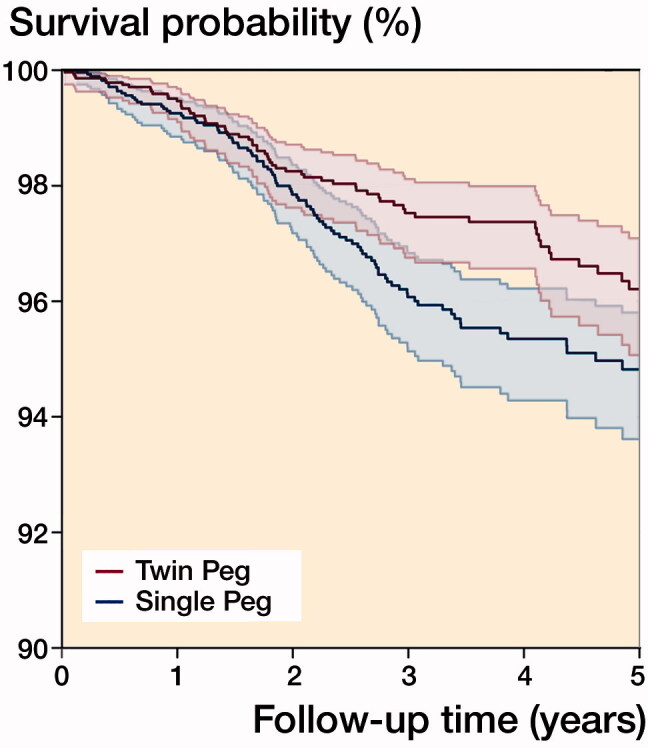
Kaplan–Meier implant survival rates for matched Single Peg (n = 2,834) and Twin Peg (n = 2,834) UKR implants up to 5 years.

In the matched cohort, the mean follow-up for both Single and Twin Peg UKRs was 3.3 (SD 2) and 3.4 years (SD 2) respectively. In total 176 knees underwent revision surgery: 102 (3.6%) Single Peg UKRs and 74 (2.6%) Twin Peg UKRs. The 5-year cumulative all-cause implant survival rates were 94.8% (CI 93.6–95.8) for Single Peg UKRs and 96.2% (CI 95.1–97.1) for Twin Peg UKRs (Figure 3). The difference in cumulative revision rates between Twin Peg and Single Peg UKRs was statistically significant (HR = 0.74, p = 0.04).

The most common reasons for revision in the Single Peg group were osteoarthritis progression (n = 26, 0.9%), pain (n = 23, 0.8%), and aseptic loosening (n = 18, 0.6%) (Table 2). In the Twin Peg UKR group the most common reasons for revision were osteoarthritis progression (n = 25, 0.9%), aseptic loosening (n = 14, 0.5%), and pain (n = 9, 0.3%) (see Table 2). There was a statistically significant (p = 0.01) difference in the revision rate for pain between the Single Peg (n = 23, 0.8%) and the Twin Peg (n = 9, 0.3%). The revision rate for femoral component aseptic loosening was significantly lower (p = 0.03) in the Twin Peg group (n = 3, 0.1%) compared with the Single Peg group (n = 11, 0.4%). However, there was no statistically significant change in the revision rate for aseptic loosening overall (Twin Peg n = 14, 0.5%; Single Peg n = 18, 0.6%; p = 0.5) or tibial component loosening (Twin Peg n = 12, 0.4%; Single Peg n = 10, 0.4%; p = 0.7).

**Table 2.** Reasons for revision in matched cohort

	Single Peg UKRs (n = 2,834)		Twin Peg UKRs (n = 2,834)		
Reasons for revision	n (%)	time (SD) **^a^**	n (%)	time (SD) **^a^**	p-value **^b^**
Aseptic loosening (any component)	18 (0.6)	2.0 (1.1)	14 (0.5)	2.9 (2.4)	0.5
Femoral component aseptic loosening ^c^	11 (0.4)	1.8 (0.8)	3 (0.1)	3.3 (2.9)	0.03
Tibial component aseptic loosening	10 (0.4)	2.1 (1.4)	12 (0.4)	3.1 (2.6)	0.7
Lysis	3 (0.1)	1.8 (0.6)	4 (0.1)	3.3 (2.9)	1.0
OA progression	26 (0.9)	2.6 (1.4)	25 (0.9)	3.1 (2.0)	0.9
Pain ^c^	23 (0.8)	2.1 (1.0)	9 (0.3)	2.6 (2.1)	0.01
Other	19 (0.7)	2.7 (1.9)	14 (0.5)	2.7 (1.8)	0.4
Dislocation subluxation revision	8 (0.3)	1.5 (1.0)	4 (0.1)	0.9 (0.6)	0.3
Instability	6 (0.2)	1.9 (1.0)	7 (0.2)	3.2 (2.9)	0.8
Component dissociation	4 (0.1)	2.1 (0.4)	3 (0.1)	2.9 (2.6)	1.0
Malalignment	8 (0.3)	1.5 (0.7)	2 (0.1)	2.1 (2.9)	0.1
Infection	7 (0.2)	1.2 (1.1)	5 (0.2)	1.1 (0.8)	0.6
Periprosthetic fracture	3 (0.1)	2.3 (3.0)	3 (0.1)	1.0 (1.0)	1.0
Wear	3 (0.1)	3.4 (1.9)	4 (0.1)	5.7 (2.7)	1.0
Stiffness	2 (0.1)	2.2 (0.2)	5 (0.2)	2.1 (1.6)	0.5
Implant fracture	1 (0)	2.0 (	0 (0)	N/A	N/A
Patellar wear	0 (0)	N/A	0 (0)	N/A	–
Tibial wear	0 (0)	N/A	0 (0)	N/A	–
Incorrect sizing	0 (0)	N/A	0 (0)	N/A	–
Patellar mal-tracking	0 (0)	N/A	0 (0)	N/A	–

**^a^** Mean time in years (standard deviation) to revision indication.

**^b^**Comparisons between the revision indications were conducted using the chi-square test or Fisher’s exact test. The latter was used in cases where the expected frequencies were < 5 in either group.

**^c^** Revision indications that were statistically significantly different in frequency between the groups.

Abbreviations: OA = osteoarthritis, UKR = unicompartmental knee replacement.

## Discussion

This is the first formal comparative observational clinical study of Single and Twin Peg cemented medial Oxford UKR femoral components. We found that the 5-year survival improved from 94.8% with the Single Peg to 96.2% with the Twin Peg and the overall revision rate decreased by 26% (p = 0.04). The main reason for this was that the revision rate for femoral component aseptic loosening (0.4% to 0.1%) and pain (0.8% to 0.3%) more than halved with the Twin Peg component. The Twin Peg was not associated with a significant increase in revision rate for any reason. This suggests that the Twin Peg femoral component is a safer component than the Single Peg.

The Twin Peg femoral component was introduced primarily to decrease the rate of femoral loosening. Our study has shown that it has reduced the rate of femoral component loosening from 11/2,834 with the Single Peg to 3/2,834 with the Twin Peg. As the incidence of loosening is low there is some uncertainly about the magnitude of the decrease but it is approximately three-quarters and it is certainly not increased. This suggests that it has improved fixation and has achieved its design aim.

The Twin Peg component was also associated with a halving in the revision rate for pain. There are various possible reasons for this. Surgeons are able to record more than 1 reason for revision, so some revisions for pain may have been cases of painful aseptic loosening where surgeons recorded both femoral loosening and pain. As femoral loosening is usually obvious at revision it is possible, but unlikely, that cases of painful early femoral loosening were just recorded as pain. With the Twin Peg there is likely to be a reduction in the incidence of anterior bearing impingement on bone, which is a potent cause of pain. The distal femur is prepared with a mill and this removes just enough bone to accommodate the Single Peg femoral component. Additional bone has to be removed in front of the milled surface to accommodate the anterior part of the bearing in full extension. We suspect that surgeons occasionally forget to remove this anterior bone in the cases where there is a fixed flexion deformity due to posterior capsule shortening, so anterior bearing impingement tends not to occur and therefore cannot be seen. Postoperatively the fixed flexion deformity steadily corrects, and impingement and pain develop. The Twin Peg component has an anterior extension to support the additional peg, which therefore cannot be inserted if the anterior bone is not removed. As a result, with the Twin Peg component surgeons cannot forget to remove the anterior bone and pain due to anterior impingement is therefore less likely.

There was no statistically significant difference in the revision rate between the Single and Twin Peg groups for any reason other than pain and femoral loosening. In particular there was no difference in the other common reasons for revision, arthritis progression and tibial loosening. More importantly there was no reason for revision that increased significantly. This suggests that the use of the 2-peg component has no downside, which is perhaps not surprising as the same operative technique, instrumentation, tibial component, and bearing are used with both Single and Twin Peg femoral components. Before our study there were no clinical studies comparing Single Peg and Twin Peg UKR designs, with the only direct comparative studies in the literature being cadaveric (Reiner et al. 2014, 2018). Reiner et al. (2018) found that that the pull-out force from cadaveric bone, as a surrogate for fixation, was substantially higher for the Twin Peg design when compared with the Single Peg design. In another study Reiner et al. (2014) observed a trend towards less subsidence in the Twin Peg design in cadaveric bone, although this did not reach statistical significance. White et al. (2015) reported the 5-year implant survival of Twin Peg UKRs as 98% but did not have a Single Peg UKR comparative arm and therefore compared their results with other Single Peg cohorts (Luscombe et al. 2007, Pandit et al. 2011) and found no differences in implant survival or in patient-reported outcome measures between the different peg groups. However, the study (White et al. 2015) was limited by a small sample size of 249 patients with only 5 revisions, thus not allowing detailed analysis of mechanisms of failure.

The main limitation of this study is the short follow up of the Twin Peg component. Additionally, the work is based on Registry data and therefore the only outcome measure is revision. However, studies of Single (Luscombe et al. 2007, Pandit et al. 2011) and Twin Peg (White et al. 2015, Lum et al. 2016) cohorts appear to report equivalent functional outcomes. Additionally, revision reasons in the NJR are those recorded at the time of surgery even if these subsequently change. Registries can underreport revisions although there is no reason to believe this would differ between the groups. Another limitation is that, despite matching, there is potential residual confounding and matching can reduce generalizability. However, virtually all Twin Peg cases were matched, which improves generalizability. Following matching the only variable with appreciable imbalance was the year of primary surgery, which is important as technique and instrumentation improved with time. However, there were no differences in our findings when we adjusted the regression models for year of primary surgery. There was a substantial proportion of BMI data missing so we did not match on BMI. However, the BMI was well balanced between groups.

In summary, this propensity-matched registry-based study found the risk of revision of the cemented Oxford UKR was 26% less with the Twin Peg femoral component compared with the Single Peg. This was primarily because the revision rates for femoral component loosening and pain more than halved. The Twin Peg was not associated with a significant increase in revision rate for any reason. This suggests that the cemented Twin Peg femoral component should be used instead of the Single Peg design.

The authors would like to thank the patients and staff of all the hospitals in England, Wales, Northern Ireland, and Isle of Man who have contributed data to the NJR. They are grateful to the Healthcare Quality Improvement Partnership, the NJR Research Sub-Committee, and staff at the NJR Centre for facilitating this work. The views expressed represent those of the authors and do not necessarily reflect those of the National Joint Registry Steering Committee or the Healthcare Quality Improvement Partnership who do not vouch for how the information is presented. Additionally, the authors would like to thank the University of Oxford for the Henni Mester Scholarship and the Royal College of Surgeons Research Fellowship who supplied HRM with funding to undertake this research. Andrew Judge was supported by the NIHR Biomedical Research Centre at the University Hospitals Bristol NHS Foundation Trust and the University of Bristol.
